# Medication errors among Iranian emergency nurses: A systematic review

**DOI:** 10.4178/epih.e2020030

**Published:** 2020-05-13

**Authors:** Zohreh Hosseini Marznaki, Somaye Pouy, Waliu Jawula Salisu, Amir Emami Zeydi

**Affiliations:** 1 Department of Nursing, Amol Faculty of Nursing and Midwifery, Mazandaran University of Medical Sciences, Sari, Iran; 2 Student Research Committee, School of Nursing and Midwifery, Guilan University of Medical Sciences, Rasht, Iran; 3 Department of Nursing, Tamale Teaching Hospital, Tamale, Ghana; 4 Department of Medical-Surgical Nursing, Nasibeh School of Nursing and Midwifery, Mazandaran University of Medical Sciences, Sari, Iran

**Keywords:** Medication errors, Nurse, Emergency service, Hospital, Iran

## Abstract

**OBJECTIVES:**

Medication errors (MEs) made by nurses are the most common errors in emergency departments (EDs). Identifying the factors responsible for MEs is crucial in designing optimal strategies for reducing such occurrences. The present study aimed to review the literature describing the prevalence and factors affecting MEs among emergency ward nurses in Iran.

**METHODS:**

We searched electronic databases, including the Scientific Information Database, PubMed, Cochrane Library, Web of Science, Scopus, and Google Scholar, for scientific studies conducted among emergency ward nurses in Iran. The studies were restricted to full-text, peer-reviewed studies published from inception to December 2019, in the Persian and English languages, that evaluated MEs among emergency ward nurses in Iran.

**RESULTS:**

Eight studies met the inclusion criteria. Most of the nurses (58.9%) had committed MEs only once. The overall mean rate of MEs was 46.2%, and errors made during drug administration accounted for 41.7% of MEs. The most common type of administration error was drug omission (17.8%), followed by administering drugs at the wrong time (17.5%) and at an incorrect dosage (10.6%). The lack of an adequate nursing workforce during shifts and improper nurse-patient ratios were the most critical factors affecting the occurrence of MEs by nurses.

**CONCLUSIONS:**

Despite the increased attention on patient safety in Iran, MEs by nurses remain a significant concern in EDs. Therefore, nurse managers and policy-makers must take adequate measures to reduce the incidence of MEs and their potential negative consequences.

## INTRODUCTION

Patient safety is a critical component of the healthcare system [1], but may be hindered by several factors, including medication errors (MEs), which are among the most common mistakes that threaten patient safety [2]. Although MEs occur with substantial frequency in hospital environments, they are significantly more common in units with more severely ill patients, such as the emergency department (ED), with rates ranging from 4% to 68% [3-6]. The unpredictability and complex nature of EDs, the critical situation of most patients, and the heavy workload due to inadequate patient-nurse ratios make the ED a high-risk area for MEs [5,6].

Previous studies have suggested that most MEs made in EDs are by nurses, with higher frequencies during administration of drugs [7-9]. Nurses spend more time with patients than most other healthcare workers and play significant roles in the process of medication management and improving patient safety [10-13]. Therefore, identifying the pattern, prevalence, and factors associated with the occurrence of MEs in the EDs can help nurse managers to develop innovative, data-driven strategies to reduce the incidence of MEs and their negative consequences [14-17]. Despite the importance of MEs in EDs and the critical role of nurses in this regard [18], there is a lack of comprehensive data concerning the prevalence and associated factors of MEs among emergency ward nurses in Iran. Therefore, this study aimed to review the literature on this issue.

## MATERIALS AND METHODS

### Search strategy

In this current review, we conducted an electronic search of several databases, including the Scientific Information Database, PubMed, Cochrane Library, Web of Science, Scopus, and Google Scholar, from database inception until December 2019. The following keywords were used: “medication error,” “prescribing error,” “medication incidents,” “medication administration error,” “drug administration error,” “drug error,” “nurse,” “emergency unit,” “emergency room,” “emergency ward,” “emergency medical service,” and “Iran.” The languages of the studies were restricted to Persian and English. When searching the Persian electronic databases, the equivalents of the keywords in Persian were used. We excluded the gray literature because, in our opinion, research in the gray literature usually does not portray the whole picture of the results, and when fully published, the results may change substantially.

### Inclusion/exclusion criteria

Full-text, peer-reviewed published studies that evaluated MEs among emergency ward nurses in Iran were included in this study. We excluded studies that were conducted among any other healthcare providers, or among nurses who worked in other, non-emergency wards. Reviews, letters, randomized controlled trials, case studies, conference papers, opinions, dissertations, reports, and editorial papers were excluded. We also excluded studies with no access to the full-text.

### Study selection

After removing duplicate studies, 2 reviewers independently evaluated the titles, abstracts, and then the full-text of studies that were potentially eligible for this review. The references of included studies were manually checked to ensure that relevant studies were not omitted. Any disagreements between reviewers were resolved by discussion.

### Data extraction and quality assessment

We designed a data extraction form to capture the following information from the included studies: name of the first author, year of the study, place of the study, sample size, age, work experience, key findings, factors affecting MEs, and the non-reporting rate of MEs by nurses. The British Sociological Association Medical Sociology Group appraisal tool was used to assess the methodological quality of the included studies [19]. Quality was categorized as high (score, 6-7), moderate (score, 3-5), or low (score, 1-2). Two independent reviewers carried out these procedures. Disagreements were resolved by discussion with a third author. To calculate the total prevalence of MEs, we used the simple mean of the sum of the ME rates and divided it by the total number of studies. We contacted the authors of included papers for additional related information if data provided were insufficient.

### Ethics statement

As the present study was a systematic review, no ethics statement was needed.

## RESULTS

### Study characteristics

**Figure 1.**
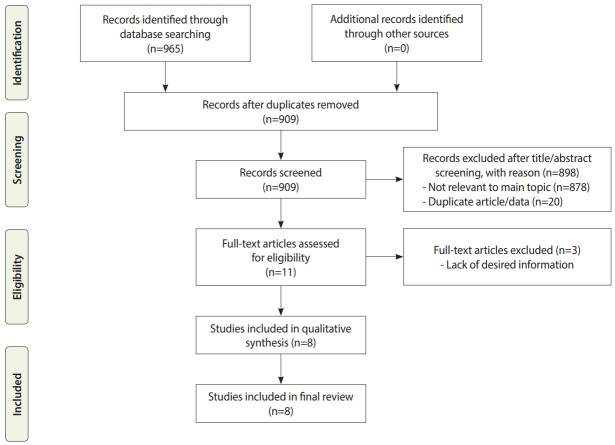
Flow diagram of the literature search.

The literature search generated 909 articles, of which 901 did not meet the inclusion criteria. The remaining 8 studies were finally included for further review (Figure 1). In total, 1,116 nurses were evaluated in the included studies, with a mean age of 32.24±6.21 years. The mean work experience of the nurses was 5.22±4.10 years. Approximately 66.0% of the nurses were women, and 55.6% were married. According to the available data, most of the nurses had committed MEs only once (58.7%), and the majority of nurses (91.4%) having a bachelor’s degree. Details of the included studies are shown in Table 1.

### Methodological quality of the included studies

All the included studies used a cross-sectional design. The quality assessment of included studies indicated that 62.0% of the studies were of high quality, and the other 38.0% were of moderate methodological quality (Table 2).

### Prevalence and types of medication errors

The overall mean rate of self-reported MEs by Iranian emergency nurses was 46.2%. According to available reported data, MEs were most commonly reported to occur during the drug administration stage, with a rate of 41.7%. The most common type of administration error was a wrong infusion rate (33.3%), followed by drug omission (17.8%), administering drugs at the wrong time (17.5%), an incorrect dosage (10.6%) and improperly administering unauthorized medications (6.4%) [2,14,18]. Another form of an incorrect dosage involves an erroneous calculation of the dose. Anticoagulants and thrombolytic medications (41.2%), antimicrobial agents (37.7%), and insulin (7.4%) were the most common drugs that were incorrectly administered [11]. Some lookalike MEs occurred among emergency nurses due to their similar names and pronunciations. For example, atropine and heparin, ceftriaxone and cefazolin, and meropenem and imipenem caused confusion. For fluids, confusion frequently occurred between normal saline and dextrose saline [16]. The non-reporting rate of MEs by emergency nurses is presented in Table 1.

### Medication error–related adverse events

Most of the included studies neither evaluated nor reported the clinical consequences of MEs and ME-related adverse events. However, 1 study stated that 97.5% of patients did not experience any critical adverse events caused by MEs made by nurses [2].

### Factors contributing to medication errors

The lack of an adequate nursing workforce during shifts and inappropriate nurse-patient ratios [6,11,16,17], as well as inadequate knowledge of medications before administering them to patients [2,16-18], accounted for the majority of reported cases of MEs made by emergency ward nurses in Iran. Demographic factors such as nurses’ age, gender, and work experience have been reported to be closely related to the risk of MEs [11-13,17]. Other factors, such as the busy nature of emergency wards [7,16] and managerial lapses, were also responsible for MEs made by nurses in EDs [14,18].

## DISCUSSION

As one of the most significant problem in hospitals, MEs have a considerable negative impact on most countries’ healthcare sectors [20-22]. MEs are responsible for the vast majority of iatrogenic injuries [23], prompting healthcare officials around the world to search for effective ways to minimize their occurrence [8,24,25]. Based on the results of the present study, the rate of drug administration errors was high (41.7%). Similarly, a previous study reported that the overall rate of MEs in Iran ranged from 14.3% to 70.0% [26]. Errors such as incorrect timing, omissions, and wrong dosages are linked to inadequate staffing, which imposes a heavy work burden on nurses [2,7,11,17,18,27]. Work overload is known to lead to fatigue [5,28], resulting in a loss of focus that consequently increases the likelihood of errors and adverse events [26,29]. We found that professional experience and education contributed significantly to reducing MEs. Nurses with higher work experience or adequate in-service training were less likely to commit MEs than less experienced nurses [30]. A study by Tang et al. [31] in Taiwan showed that nurses’ inadequate training in the wards was associated with an increased risk of MEs. Another study conducted in Canada showed that insufficient training of the staff contributed to the incidence of MEs, although to a minor extent [32]. Indeed, training has direct advantages, such as augmenting nurses’ level of knowledge and skills and improving the quality of nursing care through the application of the learned knowledge [33]. Thus, frequent in-service training for nurses is beneficial because it increases nurses’ knowledge. Other forms of MEs, such as dispensing errors, which involve dispensing medications that vary from the written orders of prescribers [34], were less widely reported in the current study, primarily because in Iran, dispensing medications is the responsibility of the pharmacist. However, the rates of dispensing and prescribing errors among other health professionals in Iran have been documented elsewhere [26].

We found that the most frequent type of ME was improper administration of medications, including omissions. According to a study by Zeraatchi et al. [11], one of the main reasons for this type of error is the use of the traditional paper-based prescribing systems rather than computerized prescription systems. The former system makes it easy for nurses to misinterpret prescriptions and administer them incorrectly. To minimize this problem, many countries have adopted computerized medical record-keeping, drug compliance systems, bar code systems, drug dispensing systems, and smart pumps to enhance the safety of pharmaceutical processes [35]. Studies have also reported that the presence of clinical pharmacists in hospital wards is one of the best-proven solutions for reducing MEs [17,36]. Zarif-Yeganeh et al. [37] reported that clinical pharmacists in EDs provided accurate drug information and monitored drug distribution, leading to a reduced incidence of MEs. The multidisciplinary team approach proposed by the Agency for Healthcare Research and Quality could serve as a dependable and applicable framework. This approach involves engaging a multidisciplinary team including advanced practice providers, physicians, pharmacists, and nurses to improve medication administration practices and patient safety [38]. However, nurses must ensure that they adhere to the 5 basic “rights” at all times when administering medications: the right patient, the right drug, the right route, the right time, and the right medication. These are effective ways to minimize MEs [39].

Based on the results of this study, it is apparent that MEs by emergency nurses are common occurrences in Iran. However, many go unreported, as suggested by other researchers [18]. Previous studies have indicated that the non-reporting of MEs is mainly due to the fear of consequences, managerial issues such as bureaucracies, and the inappropriate response of managers [26,40]. The lack of accurate information on MEs in Iran could be a sign of a weakness in the system for reporting MEs [26]. Therefore, measures such as the implementation of supportive and non-punitive systems, along with regular training, will help medical staff understand that not reporting errors imposes more significant damages than reporting them [41]. Nurses must be well informed about what constitutes an error, how errors occur, which incidents should be reported, and clear-cut reporting channels [42]. With these measures in place, the rate of MEs could be reduced. Furthermore, managers and decision-makers in the field of nursing should provide appropriate conditions to reduce the incidence of MEs in EDs in Iran. For example, regular training should be organized for the nursing staff, while ensuring that the staffing level matches patient numbers. Doing so will reduce staff workload and fatigue, which are contributors to MEs. Regular training is recommended to keep nurses updated about new medications and drug-administration protocols [43]. Furthermore, nurse authorities and hospital managers should endeavor to established computerized prescribing systems in all hospital units [44]. Computerized systems are effective at carrying out orders and detecting errors, and are not susceptible to basic human shortfalls such as fatigue and forgetfulness [45]. Similarly, computerized systems perform adequate checks, and are effective countermeasures against errors resulting from prescribers’ ineligible handwriting and confusions relating to similar drug names and unclear abbreviations [42]. A limitation of this study is that since it focused on a sensitive topic, the findings of some of the included studies might not have been fully accurate, because nurses may fear the negative consequences of reporting MEs, such as punishments, legal problems, and punitive organizational measures [46]. This possibility may have led to the misrepresentation of some aspects of the results presented herein.

## CONCLUSION

Despite the increased attention on patient safety in Iran, MEs made by nurses remain a significant concern in EDs. Based on our findings, nearly half of the nurses who work in EDs in Iran have made MEs of some type during practice. Inappropriate nurse-patient ratios and nurses’ lack of adequate knowledge about medications are some of the critical factors responsible for MEs. Nurse managers and policy-makers must implement effective measures to reduce the incidence of MEs and its potential negative consequences.

## Figures and Tables

**Table 1.** Basic characteristics and key findings of the included studies

Study	Place	Sample size	Age, mean±SD (yr)	Work experience, mean±SD or %, (yr)	Key findings	Factors affecting MEs	Non-reporting rate of MEs (%)
Mosakazemi et al., 2019 [16]	Shiraz	106 nurses	27.00±4.58	≤5: 78.3	72.6% of nurses reported at least one error in using drugs with a similar appearance; The most common mistakes in lookalike medication were related to ampoules (heparin and atropine) and vials (ceftriaxone and cefazolin, meropenem and imipenem); The rate of lookalike MEs showed statistically significant relationships with marital status (single>married), work experience (lower in nurses with higher work experience), and age (a reduction in the error rate with increasing age); The overall rate of ME by nurses was 72.6%.	Low nurse-to-patient ratio, extra work followed by excessive fatigue; overcrowding	89.4
				5-10: 14.2			
				>10: 7.5			
Izadpanah et al., 2018 [17]	Tehran	423 nurses	32.5, ranging, 23-49	≥5: 138 (32.6); <5: 285 (67.4)	Administration of the drugs at the wrong time, using an incorrect technique of administration, wrong dosage, forgetting the dosage of the drug, administering additional doses, and administering the drug to the wrong patient were the most common types of MEs; The total number of monthly self-reported MEs was 41.9; The overall rate of ME by nurses was 41.9%	Shift work, illegible physician orders, shortage of workforce, high workload, incomplete physician orders; use of lookalike and sound-alike drugs, absence of pharmacist/pharmaceutical experts, lack of adequate training regarding drug therapy	ND
Rezaei Farsani et al., 2017 [13]	Shahrkord	221 nurses	Most of the nurses were 26-30	>30% had 5-10 of work experience	The mean rate of MEs was 12.48 per nurse; the occurrence of MEs was significantly higher among men and evening and night shift nurses; The overall rate of ME was 33.5%.	High workload, shortage of nurses; fatigue resulting from excessive work, high workload, shift length, lack of adequate time; insufficient pay	ND
Vazin et al., 2014 [6]	Shiraz	202 nurses	53.00±18.17	5.0±4.7	The highest frequency of errors was related to cardiovascular (27.2%) and antimicrobial (23.6%) medications; Most medications were administered orally (54.5%) and through an intravenous infusion (27.7%); The highest rate of errors occurred during the administration phase (37.6%), followed by errors of prescription (21.1%) and transcription (10%); Omission (7.6%) and wrong timing (4.4%) were the most frequent administration errors; The overall rate of ME by nurses was 68.6%	Less-experienced nurses, higher patient-to-nurse ratio, morning shifts, shortage of nurses	31.5
Mirzaei-Alavijeh et al., 2014 [12]	Kermanshah	70 nurses	29.70±6.61	6.98±6.04	22.4% of the participants had a history of MEs at least once; Logistic regression showed that sex (OR, 1.471, p=0.035) and job history (OR, 1.695, p=0.084) could predict MEs; The overall rate of ME by nurses was 22.4%	Work environment, job experience; knowledge and skill, job history, gender	ND
Ehsani et al., 2013 [2]	Tehran	94 nurses	27.70 ± 3.40	7.3 ± 3.4	72% of nurses did not report MEs; The most common types of MEs made by nurses were a wrong infusion rate (33.3%) and incorrect drug dosage (23.8%); The most common reasons for refusing to report MEs were fear of its harmful effects, such as a low fee for service (50.0%) and legal consequences (42.8%), inappropriate or negative attitude of managers toward reporting errors (40%) and the feeling that it is unimportant to report from the nurses’ perspective (38%); The overall rate of ME by nurses was 46.8%	Lack of sufficient pharmacological information, using abbreviated names of drugs, similarities among drug names, fatigue resulting from hard work	72.7
Zeraatchi et al., 2013 [11]	Tehran	500 patients	52.9±18.0 (patients’ age)	ND	39.2% of MEs were made by nurses; The most common MEs by nurses during drug administration were omission errors (16.2%) followed by unauthorized drug administration (6.4%); Most of the MEs occurred while nurses were administering anticoagulants and thrombolytics (41.2%), followed by antimicrobial agents (37.7%) and insulin (7.4%); The overall rate of ME by nurses was 39.2%	Work shift/shift length, day of the week, inexperience, lack of supervision	ND
Dabaghzadeh et al., 2013 [7]	Tehran	275 patients	ND	ND	44.5% of MEs in the ED were made by nurses and occurred during the administration of drugs (63.6%); No relationship was found between gender and stage of errors; The most common MEs were omission (29.6%), prescribing errors (22.6%), and administration of the incorrect dose (11.2%); The overall rate of ME by nurses was 44.5%	Older staff beyond age 50 were more likely to make MEs, high workload, understaffing	25.1

ME, medication error; ND, no data; ED, emergency department; OR, odds ratio.

**Table 2.** Methodological quality assessment of the included studies.

Quality assessment	Reference							
	[13]	[17]	[16]	[6]	[12]	[2]	[11]	[7]
1. Appropriate research desigh (Y/N)	Y	Y	Y	Y	Y	Y	Y	Y
2. Appropriate recruitment strategy (Y/N)	N	Y	Y	Y	Y	Y	Y	Y
3. Response rate reported (Y/N)	Y	Y	Y	Y	Y	Y	Y	Y
4. Sample representative of similar population (Y/N)	Y	Y	Y	Y	Y	Y	Y	Y
5. Objective and reliable measures used (Y/N)	N	N	N	N	N	N	N	N
6. Power calculation/justification of numbers reported (Y/N)	N	N	Y	Y	Y	Y	Y	N
7. Appropriate statistical analysis (Y/N)	N	Y	Y	Y	Y	Y	Y	Y
Quality indicators met (out of 7)	3	5	6	6	6	6	6	5

Y, yes; N, no.

## References

[1] Acheampong F, Anto BP, Koffuor GA (2014). Medication safety strategies in hospitals--a systematic review. Int J Risk Saf Med.

[2] Ehsani SR, Cheraghi MA, Nejati A, Salari A, Esmaeilpoor AH, Nejad EM (2013). Medication errors of nurses in the emergency department. J Med Ethics Hist Med.

[3] Hillin E, Hicks RW (2010). Medication errors from an emergency room setting: safety solutions for nurses. Crit Care Nurs Clin North Am.

[4] Shitu Z, Aung MM, Tuan Kamauzaman TH, Ab Rahman AF (2020). Prevalence and characteristics of medication errors at an emergency department of a teaching hospital in Malaysia. BMC Health Serv Res.

[5] Zarea K, Mohammadi A, Beiranvand S, Hassani F, Baraz S (2018). Iranian nurses’ medication errors: a survey of the types, the causes, and the related factors. Int J Afr Nurs Sci.

[6] Vazin A, Zamani Z, Hatam N (2014). Frequency of medication errors in an emergency department of a large teaching hospital in southern Iran. Drug Healthc Patient Saf.

[7] Dabaghzadeh F, Rashidian A, Torkamandi H, Alahyari S, Hanafi S, Farsaei S (2013). Medication errors in an emergency department in a large teaching hospital in Tehran. Iran J Pharm Res.

[8] Shahrokhi A, Ebrahimpour F, Ghodousi A (2013). Factors effective on medication errors: a nursing view. J Res Pharm Pract.

[9] Patanwala AE, Warholak TL, Sanders AB, Erstad BL (2010). A prospective observational study of medication errors in a tertiary care emergency department. Ann Emerg Med.

[10] Rossetti AC, Gaidzinski RR, Fugulin FM (2013). Nursing workload in the emergency department: a methodological proposal. Rev Lat Am Enfermagem.

[11] Zeraatchi A, Talebian MT, Nejati A, Dashti-Khavidaki S (2013). Frequency and types of the medication errors in an academic emergency department in Iran: the emergent need for clinical pharmacy services in emergency departments. J Res Pharm Pract.

[12] Mirzaei-Alavijeh M, Jalilian F, Karami-Matin B, Ghaderi A, Mahboubi M, Janizadeh R (2014). Needle-stick and medication errors in emergency nurses are due to their job stresses? A descriptive study in Kermanshah Hospitals, Iran. J Biol Today’s World.

[13] Rezaei Farsani M, Farokhpour M (2017). Study of the rate, type and factors affecting drug errors from the perspective of nurses working in intensive care and emergency unite of educational hospitals of Shahrekord University of Medical Sciences. Dev Strategies Med Educ.

[14] Salavati S, Hatamvand F, Tabesh H (2012). Nurses’ perspectives on causes of medication errors and non-reporting at ED. Iran J Nurs.

[15] Di Simone E, Giannetta N, Auddino F, Cicotto A, Grilli D, Di Muzio M (2018). Medication errors in the emergency department: knowledge, attitude, behavior, and training needs of nurses. Indian J Crit Care Med.

[16] Mosakazemi SZ, Bastani P, Marzaleh MA, Peyravi MR (2019). A survey on the frequency of medication errors caused due to look-alike drugs in the emergency department of the educational hospitals of Shiraz, Iran, 2016. Iran J Health Saf Environ.

[17] Izadpanah F, Nikfar S, Bakhshi Imcheh F, Amini M, Zargaran M (2018). Assessment of frequency and causes of medication errors in pediatrics and emergency wards of teaching hospitals affiliated to Tehran University of Medical Sciences (24 hospitals). J Med Life.

[18] MohammadNejad E, Ehsani SR, Salari A, Sajjadi A, Hajiesmaeel-Pour A (2013). Refusal in reporting medication errors from the perspective of nurses in emergency ward. J Res Dev Nurs Midwifery.

[19] Blaxter M (1996). Criteria for the evaluation of qualitative research papers. Med Sociol News.

[20] Keers RN, Williams SD, Cooke J, Ashcroft DM (2013). Prevalence and nature of medication administration errors in health care settings: a systematic review of direct observational evidence. Ann Pharmacother.

[21] Salmasi S, Khan TM, Hong YH, Ming LC, Wong TW (2015). Medication errors in the southeast Asian countries: a systematic review. PLoS One.

[22] Sherriff K, Wallis M, Burston S (2011). Medication calculation competencies for registered nurses: a literature review. Aust J Adv Nurs.

[23] Armitage G, Newell R, Wright J (2010). Improving the quality of drug error reporting. J Eval Clin Pract.

[24] Anderson S (2002). The state of the world’s pharmacy: a portrait of the pharmacy profession. J Interprof Care.

[25] Hayes C, Jackson D, Davidson PM, Power T (2015). Medication errors in hospitals: a literature review of disruptions to nursing practice during medication administration. J Clin Nurs.

[26] Mansouri A, Ahmadvand A, Hadjibabaie M, Javadi M, Khoee SH, Dastan F (2014). A review of medication errors in iran: sources, underreporting reasons and preventive measures. Iran J Pharm Res.

[27] Brown M (2005). Medication safety issues in the emergency department. Crit Care Nurs Clin North Am.

[28] Cramer H, Pohlabeln H, Habermann M (2013). Factors causing or influencing nursing errors as perceived by nurses: findings of a cross-sectional study in German nursing homes and hospitals. J Public Health.

[29] Gholipour Baradari A, Hoseini SH, Zamani Kiasari A, Ala SH, Emami Zeydi A, Mahdavi A, Mirbakhshi SF (2013). Effect of zinc supplement on job stress of ICU nurses. J Babol Univ Med Sci.

[30] Chang Y, Mark B (2011). Effects of learning climate and registered nurse staffing on medication errors. J Nurs Adm.

[31] Tang FI, Sheu SJ, Yu S, Wei IL, Chen CH (2007). Nurses relate the contributing factors involved in medication errors. J Clin Nurs.

[32] Sears K, O’Brien-Pallas L, Stevens B, Murphy GT (2013). The relationship between the nursing work environment and the occurrence of reported paediatric medication administration errors: a pan Canadian study. J Pediatr Nurs.

[33] Qalehsari MQ, Khaghanizadeh M, Ebadi A (2017). Lifelong learning strategies in nursing: a systematic review. Electron Physician.

[34] Cheung KC, Bouvy ML, De Smet PA (2009). Medication errors: the importance of safe dispensing. Br J Clin Pharmacol.

[35] Frith KH, Anderson EF, Tseng F, Fong EA (2012). Nurse staffing is an important strategy to prevent medication error in community hospitals. Nurs Econ.

[36] Pahlevan D, Jandaghi J, Shaeeri M, Razavi MR, Abdollahpour A, Kermani A (2018). Classification and assessment of medication errors in the emergency unit of a hospital in Iran by SHERPA. World Fam Med.

[37] Zarif-Yeganeh M, Rastegarpanah M, Garmaroudi G, Hadjibabaie M, Sheikh Motahar Vahedi H (2017). Incidence of medication discrepancies and its predicting factors in emergency department. Iran J Public Health.

[38] Winters BD, Bharmal A, Wilson RF, Zhang A, Engineer L, Defoe D (2016). Validity of the agency for health care research and quality patient safety indicators and the centers for Medicare and Medicaid hospital-acquired conditions: a systematic review and meta-analysis. Med Care.

[39] Edwards S, Axe S (2015). The 10 ‘R’s of safe multidisciplinary drug administration. Nurs Prescr.

[40] Mirsadeghi A, Pazokian M (2015). Barriers to reporting medication errors in Iran: a systematic review. Int J Med Rev.

[41] Amrollahi M, Khanjani N, Raadabadi M, Hosseinabadi M, Mostafaee M, Samaei S (2017). Nurses’ perspectives on the reasons behind medication errors and the barriers to error reporting. Nurs Midwifery Stud.

[42] Cloete L (2015). Reducing medication errors in nursing practice. Nurs Stand.

[43] Maxwell SR, Webb DJ (2019). Improving medication safety: focus on prescribers and systems. Lancet.

[44] Mieiro DB, Oliveira ÉB, Fonseca RE, Mininel VA, Zem-Mascarenhas SH, Machado RC (2019). Strategies to minimize medication errors in emergency units: an integrative review. Rev Bras Enferm.

[45] Kenawy AS, Kett V (2019). The impact of electronic prescription on reducing medication errors in an Egyptian outpatient clinic. Int J Med Inform.

[46] Peyrovi H, Nikbakht Nasrabadi A, Valiee S (2016). Exploration of the barriers of reporting nursing errors in intensive care units: a qualitative study. J Intensive Care Soc.

